# Septic polyarthritis with *Mycoplasma salivarium* in a patient with common variable immunodeficiency: case report and review of the literature

**DOI:** 10.1099/acmi.0.000221

**Published:** 2021-04-09

**Authors:** Arthur H. Totten, Li Xiao, Donna M. Crabb, Amy E. Ratliff, Ken B. Waites, Tracy Hwangpo, T. Prescott Atkinson

**Affiliations:** ^1^​ Department of Pediatrics, University of Alabama at Birmingham, Birmingham, AL, USA; ^2^​ Department of Medicine, University of Alabama at Birmingham, Birmingham, AL, USA; ^3^​ Department of Pathology, University of Alabama at Birmingham, Birmingham, AL, USA; ^†^​ Present address: Department of Laboratory Medicine, Clinical Center, National Institutes of Health, Bethesda, MD, USA

**Keywords:** bacterial arthritis, common variable immunodeficiency, hypogammaglobulinemia, *Mycoplasma salivarium*

## Abstract

*
Mycoplasma salivarium* is a common mycoplasma usually isolated from human oropharynx, particularly from individuals with periodontal disease. It is also among the more common mycoplasmal contaminants of eukaryotic cell cultures. Although *M. salivarium* has been isolated occasionally from abscesses and other sterile sites, to our knowledge, only three cases of septic arthritis have been documented in the past due to this organism, all in patients with humoral immunodeficiency. We now report a fourth case of septic polyarthritis in a patient with profound hypoimmunoglobulinemia who had experienced dental abscesses within the preceding 2 years. Our case highlights the importance of considering invasive mycoplasmal infection in hypogammaglobulinemic patients. It is likely of significance that the patient had suffered recurrent dental abscesses as a source of infection with *M. salivarium *.

## Introduction

Common Variable Immunodeficiency (CVID) is a complex primary immunodeficiency disorder caused by defective B cell differentiation to plasma cells and a resulting defect in specific antibody responses to antigenic stimulation [1]. The syndrome can be caused by mutations in at least 19 known genes but a possible genetic basis for the disease in the majority of patients is still unknown. By definition, IgG and at least one other isotype (IgA or IgM) are less than two standard deviations below the mean for patient age. CVID is the most common serious immunoglobulin deficiency, with a prevalence of about 1 : 25 000 [2].

Disseminated infections with *Mycoplasma* and *Ureaplasma* species are known to occur in hypogammaglobulinemic patients. The most commonly described type of mycoplasma infection in such patients is septic arthritis, and case reports have regularly documented infection with *M. pneumoniae, M. hominis*, and *Ureaplasma* spp [3–8]. It can be inferred that protective antibody is particularly important in preventing dissemination of mycoplasmas from the mucosa of the respiratory and urogenital tracts.

*Mycoplasma salivarium* is a pathobiont that is present in the oral flora in up to 97 % of humans and is particularly prevalent in the periodontal sac [9–11]. Like other mycoplasmas, the organism lacks a cell wall and has a highly reduced genome (~713 526 bp) consistent with its parasitic lifestyle. Although generally considered nonpathogenic, *M. salivarium* has been increasingly implicated in invasive infections, particularly in immunocompromised or debilitated individuals. This report highlights the fact that invasive infections with mycoplasmas and ureaplasmas may have a slowly progressive, indolent clinical presentation, particularly in patients with immunodeficiency.

## Case presentation

A 32 year-old African-American female had previously presented to Children’s of Alabama at age 15 in Birmingham, Alabama with a cavitary pneumonia and was treated with clindamycin and cefuroxime intravenously for 10 days. Due to the severity of the infection, an immunologic workup was performed, and although the patient was mildly lymphopenic (absolute lymphocyte count 900 μl^−1^), the lymphocyte subset distributions were normal. Immunoglobulin levels were all below the normal ranges for age, and she was diagnosed with CVID. The patient received 2 monthly doses of intravenous IgG replacement therapy (IVIG) and recovered from her pneumonia but was subsequently lost to follow-up for 16 years.

**Fig. 1.**
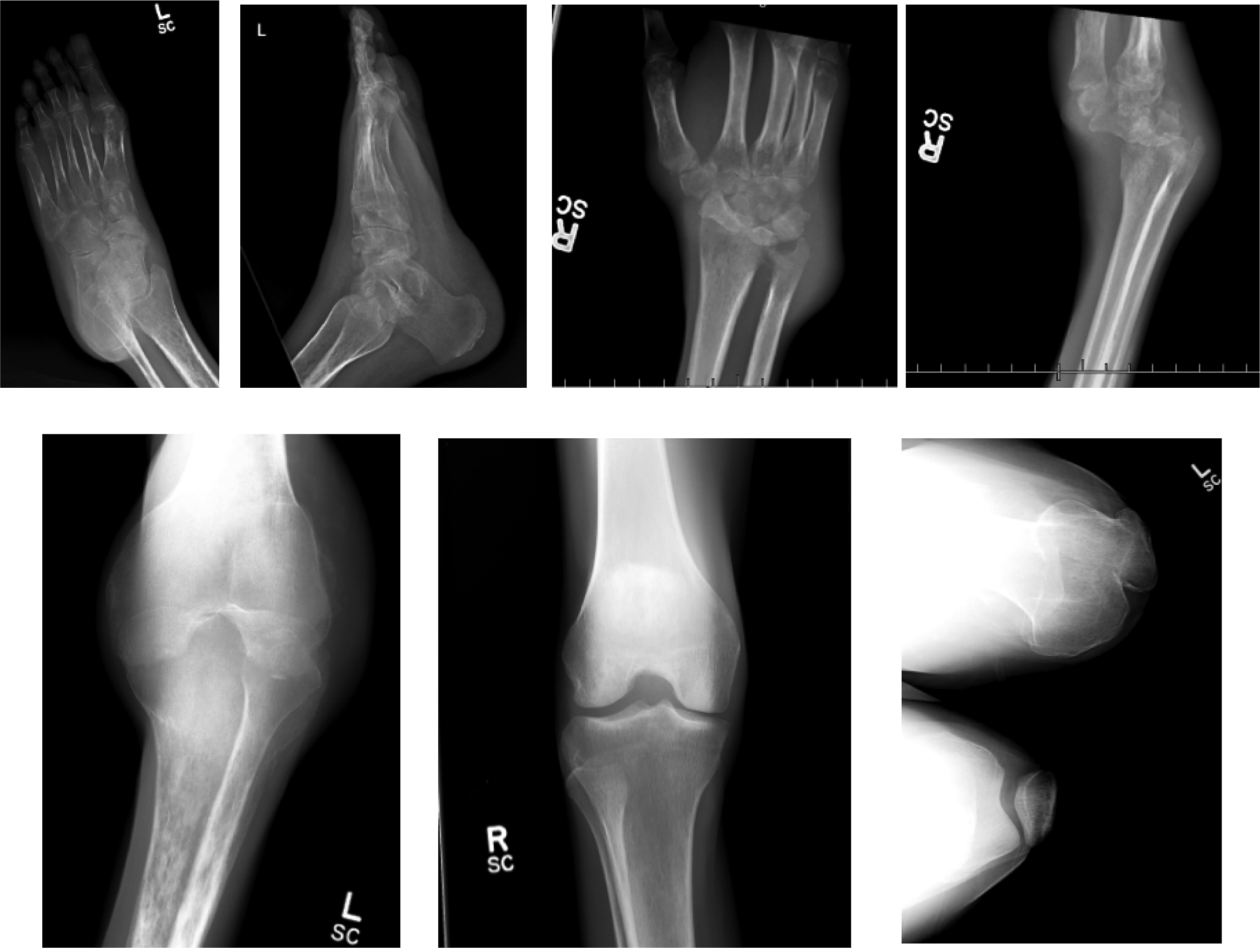
Destructive changes in left ankle, right wrist and left knee. The upper left two panels demonstrate diffuse osteopenia of the left foot and ankle with osseous destruction and loss of subchondral bone involving the talus and navicular at the talonavicular joint. The right wrist (upper two right panels) demonstrates severe osteopenia with the carpus volarly dislocated from the distal radius. There are irregular destructive changes of the distal ulna especially along the distal radioulnar joint. There is advanced osteopenia centred around the left knee (lower three panels) with loss of subchondral bone involving the patellofemoral and femorotibial articular surfaces with ossific densities in the superpatellar pouch suggesting some degree of chronicity.

At the time of her diagnosis with septic arthritis during hospital admission at 32 years of age, the patient had a history of weight loss, malaise, nausea and vomiting, diarrhoea and swelling in left knee and right wrist dating from a knee injury as detailed below. During the intervening years she had suffered chronic ear infections, chronic sinusitis, and axillary skin abscesses and had experienced several dental abscesses requiring tooth extraction and antibiotics over the preceding 2 years. She also suffered from occasional episodes of oral candidiasis/thrush. Eighteen months prior to admission, the patient had suffered trauma to the left knee and 9 months later received treatment by an orthopedist that included arthroscopy and arthrocentesis that yielded no fluid for diagnostic testing. At that time she was also beginning to have intermittent left ankle swelling and low-grade fevers. About a year after her knee injury she began experiencing pain and swelling intermittently in her left thumb and wrist. Two months prior to admission the pain and swelling in the right wrist increased significantly with the appearance of nodules around the joint. At time of admission, radiographs revealed severe destructive changes in the wrist and knee, as well as left ankle (Fig. 1). Initial aspiration of joint fluid from the knee had been nondiagnostic. On admission joint fluid obtained from the right wrist was grossly purulent (216 120 WBC μl^−1^, 77 % neutrophils, and 186 000 RBC μl^−1^). She was treated empirically with intravenous vancomycin and ceftriaxone, and doxycycline was added on hospital day 13 due to continued negative microbiologic test results and continued fevers and joint pain. She continued to run intermittent fevers as high as 103 °F, and aspiration of left knee fluid on day six of admission revealed 47 500 WBC μl^−1^ but routine microbial cultures and Gram-stain were negative from both joint aspirations. Laboratory evaluation revealed profound agammaglobulinemia (IgM <20 mg dl^−1^, range 43–215; IgA <6, range 59–371; IgG <200, range 678–1425; IgE <1 IU ml^−1^, range 6–216), and there was B cell lymphopenia noted on flow cytometric analysis of patient’s peripheral blood lymphocytes (2 %, range 6–19 %; absolute numbers 27 µl^−1^, range 100–500). Other lymphocyte populations were normal in distribution and number, and T cell antigen recall responses were also normal (Supplementary Table).

As multiple bacterial cultures remained negative for 2 weeks, patient samples were submitted for PCR of 16S rRNA domain IV-V followed by next generation sequencing (NGS) of PCR product (University of Washington, Molecular Diagnosis Microbiology Section) in an attempt to identify the presumptive microbial infection. Additionally, samples were submitted for culture to the UAB Diagnostic Mycoplasma Laboratory. Culture of joint fluid in SP4 broth and agar was performed, and large colonies with a fried-egg shape typical of mycoplasma species were observed on the agar plate after 16 days of incubation at 37 °C. Then 16S rRNA NGS analysis revealed the organism to be *M. salivarium *. Diagnosis was further confirmed on the UAB clinical isolate by PCR, amplifying the full length of the 16S RNA gene followed by Sanger sequencing.

Antimicrobial susceptibility testing (AST) was carried out on the patient’s *M. salivarium* isolate in the UAB Diagnostic Mycoplasma Laboratory to determine the minimum inhibitory concentration (MIC) of different antimicrobials by broth microdilution methods standardized for other human mycoplasma species [12]. The following MICs were obtained: levofloxacin (4 µg ml^−1^), doxycycline (0.016 µg ml^−1^), clindamycin (0.063 µg ml^−1^), and erythromycin (2 µg ml^−1^). Using MIC interpretive breakpoints for *Mycoplasma hominis *, the *M. salivarium* isolate would be considered susceptible to doxycycline, and clindamycin and resistant to levofloxacin. There are no MIC breakpoints for erythromycin for *M. hominis* since this mycoplasma is innately resistant to 14 and 15 membered macrolide antibiotics, but the erythromycin MIC of 2 µg ml^−1^ would be considered resistant using the interpretive criteria for *M. pneumoniae* [12]. On hospital day 20 her antibiotics were changed to oral moxifloxacin, 400 mg daily. At discharge after a total of 40 days hospitalization, the patient was given an extended course of oral moxifloxacin 400 mg daily over 4 months together with monthly IVIG (25 grams, 0.5 gm kg^−1^), infusions of which had been given three times during her hospitalization. The patient developed recurrent *Clostridium difficile* colitis over the course of treatment (diagnosed on hospital day 33 using a DNA molecular assay which detects the presence of the pathogenicity locus (PaLoc)), but her joint inflammation improved and the infection cleared leaving her with significant disability in the affected joints.

Over the last 6 years since she has been able to get uninterrupted monthly treatment of IVIG, she has not had any other severe infections. She has recovered some function of her wrist and was able to get a left knee total replacement 2 years ago. Recent studies showed she has nearly zero B cells in her peripheral blood, and a primary immunodeficiency panel of 207 genes including 32 associated with CVID did not find any significant variants. She has not yet had exome sequencing.

## Discussion

Although generally considered nonpathogenic, *M. salivarium* has been increasingly implicated in invasive infections, particularly in immunocompromised or debilitated individuals. Successful culture requires specialized media and expertise, and cultures in appropriate media may nevertheless take several days or weeks to turn positive. Therefore, utilization of specific nucleic acid amplification techniques or generalized 16S rRNA microbial sequencing has become increasingly common in cases of suspected disseminated mycoplasmal infection. There are no commercial nucleic acid amplification tests that can identify *M. salivarium* or distinguish it from other mycoplasma species in clinical specimens, so shotgun metagenomic sequencing and 16S rRNA PCR followed by next generation sequencing of the PCR product are currently the most efficient methods to obtain a definitive identification.

CVID is the most common serious immunoglobulin deficiency, with a prevalence of about 1 : 25 000 [2]. Case reports have regularly documented joint infections in CVID patients as well as other patients with antibody deficiencies by *M. pneumoniae *, *M. hominis *, and *Ureaplasma* spp. All three previously published cases of *M. salivarium* arthritis occurred in antibody deficient patients, two with a history of B cell lymphoma or leukaemia, one of which occurred as a post-operative infection in a prosthetic joint, and one with primary immunodeficiency [13–15]. It can be inferred that protective antibody is particularly important in preventing dissemination of mycoplasmas from the mucosa of the respiratory and urogenital tracts.

Septic arthritis is a relatively common type of infection which can arise through hematogenous dissemination, trauma, spread from a periarticular tissue infection, or postoperative infection of a prosthesis [16]. As illustrated by our patient, joint infections by mollicutes can be deceptively slow-moving, with lower joint fluid cell counts and relatively reduced signs of joint inflammation that can lull the unsuspecting clinician into attempting to treat a presumptive mechanical or rheumatologic joint process until joint destruction is far advanced. During the slow progression of her joint infections, our patient had been evaluated by a rheumatologist and a tentative diagnosis of psoriatic arthritis was being considered. There is little doubt that hematogenous dissemination of *M. salivarium* to multiple joints occurred in this severely hypoimmunoglobulinemic patient, and this may have occurred in association with procedures for her dental abscesses. A similar infection has been reported for the periodontal pathobiont *Porphyromonas gingivalis* following a dental procedure [17].

Culture of mycoplasmas is difficult even with the highly enriched media that have been developed specifically for their isolation. A recent report documented that the addition of catalase to the culture medium augmented the growth of *M. pneumoniae* [18], which like *M. salivarium* produces large amounts of H_2_O_2_ that can inhibit the growth of the organism [19]. The patient’s *M. salivarium* isolate was cultured with and without catalase (1.5 mg ml^−1^ bovine liver catalase, 2000–5000 U mg^−1^, Sigma), and showed a three- to seven-fold increase in Colony Forming Units (c.f.u.) ml^−1^ with catalase supplementation in the SP4 growth medium compared to conventional SP4 (*P* <0.05). Additionally, cultures became positive on average a day or two earlier than those without catalase. Catalase was not added to the SP4 medium used for MIC determination. Thus, as demonstrated for *M. pneumoniae* inclusion of catalase in SP4 broth facilitated the culture of *M. salivarium *. For laboratories culturing *M. salivarium *, or other H_2_O_2_-producing mollicutes, inclusion of catalase increases bacterial growth rate and measurable density and may be beneficial for faster turn-around time for phenotypic diagnosis, but further validation studies are needed.

Successful resolution of invasive mycoplasmal infections in immunodeficient persons typically requires weeks to months of antimicrobial administration and relapses may still occur once the antimicrobial agent is discontinued. Fluoroquinolones, such as moxifloxacin, are bactericidal against *Mycoplasma* spp and are usually the best treatment alternatives for invasive infections in immunodeficient persons due to the low likelihood of resistance [20].

## Conclusion

This case highlights the importance of considering mycoplasmal infection in indolent arthritis in hypogammaglobulinemic patients and the need to obtain appropriate diagnostic studies including specialized culture for mycoplasmas and broad-range next generation sequencing to identify the species involved and obtain *in vitro* antimicrobial susceptibilities to guide treatment.

## Supplementary Data

Supplementary material 1
